# Development and validation of a nomogram to predict lymph node metastasis in patients with progressive muscle-invasive bladder cancer

**DOI:** 10.3389/fonc.2024.1342244

**Published:** 2024-05-16

**Authors:** Yi Qiao, Yuefeng Jia, Lei Luo, Bin Li, Fei Xie, Hanshu Wang, Shengxian Li

**Affiliations:** ^1^ Department of Urology, The Affiliated Hospital of Qingdao University, Qingdao, China; ^2^ Department of Andrology, The Affiliated Hospital of Qingdao University, Qingdao, China

**Keywords:** nomogram, muscle-invasive bladder cancer, lymph node metastasis, lymph node, retrospective study

## Abstract

**Purpose:**

To develop and validate a nomogram for preoperative prediction of lymph node metastasis in patients with progressive muscle-invasive bladder cancer.

**Materials and methods:**

We retrospectively recruited patients, divided them into training and validation cohorts, and gathered patient demographics, pathology data of transurethral bladder tumor resection specimens, imaging findings, and laboratory information. We performed logistic regression analyses, both single-variable and multi-variable, to investigate independent preoperative risk variables and develop a nomogram. Both internal and external validations were conducted to evaluate the predictive performance of this nomogram.

**Results:**

The training cohort consisted of 144 patients with advanced muscle-invasive bladder cancer, while the validation cohort included 62 individuals. The independent preoperative risk factors identified were tumor pathology grade, platelet count, tumor size on imaging, and lymph node size, which were utilized to develop the nomogram. The model demonstrated high predictive accuracy, as evidenced by the area under the receiver operating characteristic curve values of 0.898 and 0.843 for the primary and external validation cohorts, respectively. Calibration curves and decision curve analysis showed a good performance of the nomogram in both cohorts, indicating its high clinical applicability.

**Conclusion:**

A nomogram for preoperative prediction of lymph node metastasis in patients with advanced muscle-invasive bladder cancer was successfully developed; its accuracy, reliability, and clinical value were demonstrated. This new tool would facilitate better clinical decisions regarding whether to perform complete lymph node dissection in cases of radical cystectomy.

## 1 Introduction

Bladder cancer (BC) is the sixth most common malignant tumor worldwide, and the ninth leading cause of malignant tumor-related deaths, owing to its high incidence and poor prognosis (1). BC is categorized into two subgroups, namely non-muscle-invasive bladder cancer (NMIBC) and muscle-invasive bladder cancer (MIBC), based on the degree of tumor infiltration into the bladder wall. Approximately 8% of patients diagnosed with NMIBC and 25% of those diagnosed with MIBC experience lymph node metastasis (LNM) (2). The presence of LNM in patients diagnosed with BC is a prognostic factor, indicating a higher likelihood of poor outcomes and aggressive tumor behavior (3). During initial diagnosis, approximately 75–85% of instances of BC are classified as non-muscle-invasive, specifically categorized as pTa, pT1, or carcinoma *in situ* (CIS) (4), while another 25% are first diagnosed with MIBC, which is referred to as primary muscle-invasive bladder cancer (PMIBC) (5). The conventional therapy for early-stage NMIBC comprises transurethral resection of bladder tumors (TURBT) followed by postoperative bladder instillation chemotherapy. Recurrence is observed in an estimated range of 30–80% of cases, whereas progression to muscle invasion occurs in 1–45% of cases within a span of 5 years (6). Therefore, individuals diagnosed with NMIBC treated with any non-MIBC therapy (TURBT, intracellular therapy) that progressed to MIBC during a later stage of observation are classified as having progressive MIBC (5, 7, 8).

According to a report published by the Bladder Cancer Research Alliance, a total of 162 patients diagnosed with clinical T1G3 disease and who underwent bladder resection were included, of which 30 individuals, accounting for 19% of the cohort, had died due to BC (9). In 50% of these patients, despite undergoing bladder resection in the early stages of disease progression, 17% showed lymph node involvement (9). Previous studies have demonstrated a limited timeframe within which patients diagnosed with high-risk or T1G3 NMIBC, who have previously undergone bladder resection and experienced therapy failure with Bacillus Calmette-Guérin (BCG), showed positive prognosis (10). Progressive BC is a significant adverse prognostic outcome of high-risk NMIBC. Therefore, new indicators for predicting LNM in patients with progressive MIBC are urgently needed.

This study utilized demographic information, pathological characteristics of TURBT specimens, imaging data, and laboratory measurements to identify potential risk variables associated with LNM in patients with progressive MIBC. Additionally, we developed a nomogram for predicting the likelihood of LNM occurrence in patients with progressive MIBC.

## 2 Materials and methods

### 2.1 Study population

The patient data utilized in this study underwent a thorough review and de-identification process by the Institutional Ethics Committee at our center. Our study included a cohort of 204 consecutive patients diagnosed with BC who received treatment from January 2012 to June 2022.

The selection of participants was based on the following predefined inclusion criteria: (i) patients diagnosed with NMIBC at Qingdao University Affiliated Hospital (Qingdao, Shandong Province, China) during the specified period, who underwent TURBT following the guidelines of the European Association of Urology (6) and subsequently received laparoscopic RC with extended pelvic lymph node dissection (PLND) up to the level of the aortic bifurcation, due to disease recurrence or progression, and had pathologically-confirmed urothelial carcinoma; (ii) patients who had had pelvic computed tomography (CT)-enhanced imaging performed within 20 days prior to their surgeries; (iii) those for whom clinical characteristics information was available; (iv) patients who received intravesical instillation therapy with pirarubicin following TURBT. The exclusion criteria encompassed two categories: (i) patients who had undergone preoperative treatment, specifically neoadjuvant chemotherapy or radiation therapy; (ii) and patients who had other concomitant malignant conditions.

The participants were categorized into two cohorts, namely a training cohort, comprising 144 individuals who received treatment from January 2012 to June 2019, and a validation cohort, comprising 62 individuals who received treatment from July 2019 to June 2022. All surgeries were performed by trained and experienced surgeons at our medical center. Pathological assessments were conducted by specialized uropathologists using multiple specimens. Pathological grades were categorized into Low-grade urothelial carcinoma (LGPUC) and High-grade urothelial carcinoma (HGPUC) based on the histological characteristics outlined in the grading system for bladder and urinary tract urothelial carcinoma malignancy published by the World Health Organization in 2004.

### 2.2 Statistical Analysis

The multivariate logistic regression analysis employed forward stepwise selection. Nomograms, receiver operator characteristics (ROC) curves, calibration curves, and decision curve analysis (DCA) were generated using the “calibrate” (11), “glmnet” (12),”rms”, “pROC”, and “rmda” programs in the R software. The statistical analyses were conducted using SPSS version 24.0 and R version 4.1.0. Statistically significant results were defined as having two-tailed p-values of < 0.05.

## 3 Results

### 3.1 Independent risk factors for lymph node metastasis and nomogram construction

**Figure 1:**
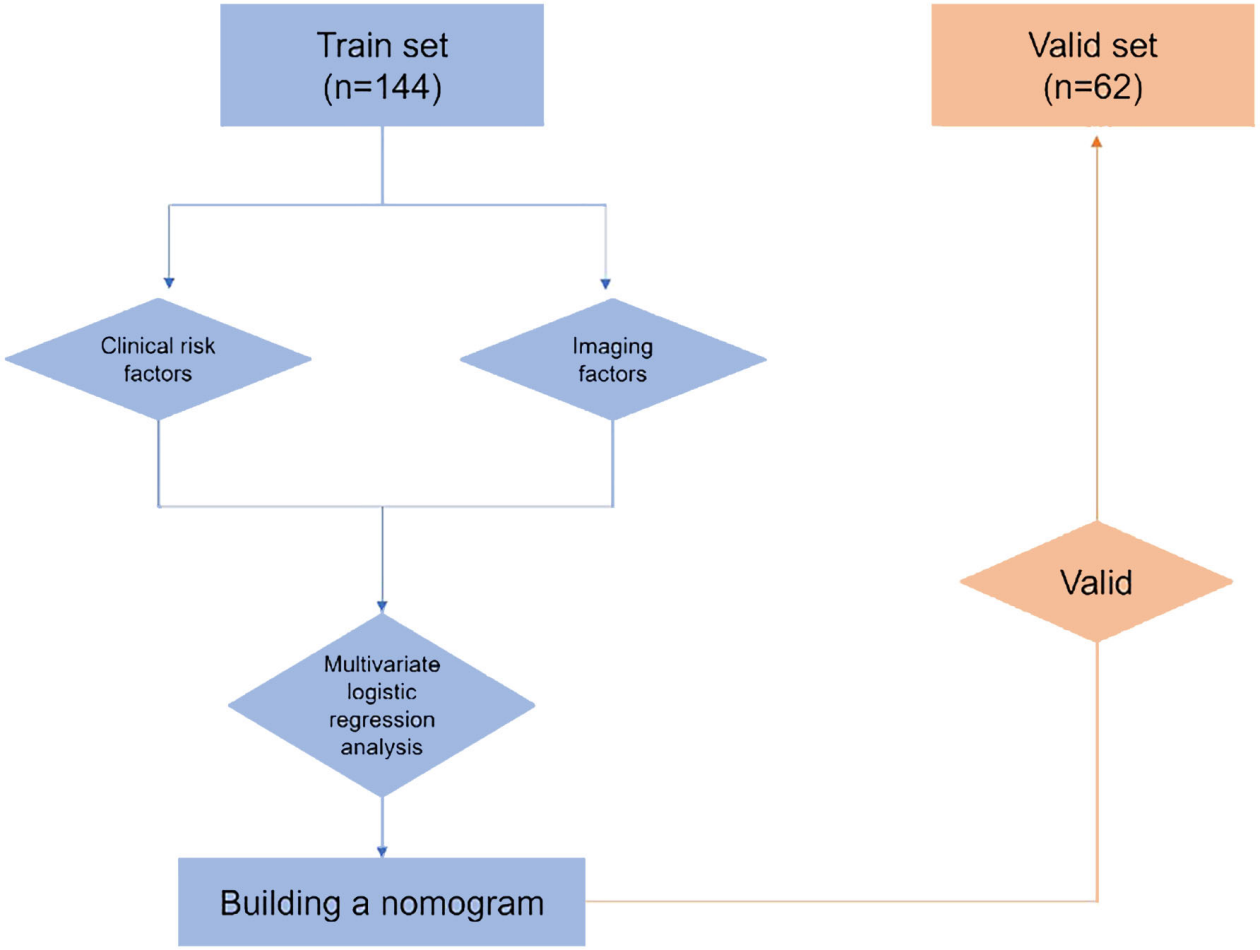
Study Flowchart.

The research workflow is depicted in **Figure 1**. **Table 1** lists the patient characteristics of those in the training and validation sets. No notable disparities were observed in the baseline characteristics between the training and validation groups. Univariate and multivariate logistic regression analyses were employed to identify the independent risk factors associated with LNM. **Table 2** lists the independent risk factors for LNM that demonstrated significant differences between patients with progressive MIBC and LNM and those without LNM, as determined by our univariate analysis. These risk factors, along with the corresponding parameters required for constructing the nomogram, were subsequently incorporated into our binary multivariate logistic regression analysis. The aforementioned parameters encompassed tumor size, lymph node size, and tumor grading.

**Table 1:** Baseline characteristics of the training and validation sets.

Variable	Total (n = 206)	Training set (n = 144)	Validation set (n = 62)	Statistic	p
Age (years)	68.73 ± 9.56	68.61 ± 9.42	69.00 ± 9.96	t = -0.267	0.790
Weight (kg)	66.34 ± 10.42	66.07 ± 10.61	66.95 ± 10.02	t = -0.553	0.581
BMI (kg/m^2^)	23.50 (21.20–25.40)	23.30 (21.25–25.60)	23.70 (21.35–25.23)	Z = 0.238	0.812
Tumor size (cm)	29.00 (18.23–44.00)	29.00 (19.75–44.00)	30.50 (15.95–44.00)	Z = 0.265	0.791
Serum calcium (mmol/L)	2.25 (2.19–2.34)	2.24 (2.18–2.32)	2.27 (2.20–2.37)	Z = 1.575	0.115
Albumin (g/L)	40.27 (37.59–43.20)	39.80 (36.89–42.59)	41.40 (38.77–44.50)	Z = 2.474	0.013
Neutrophil count (mmol/L)	4.14 (3.16–5.45)	4.11 (3.23–5.50)	4.16 (3.16–5.30)	Z = 0.182	0.855
Lymphocyte count (mmol/L)	1.81 (1.31–2.23)	1.75 (1.31–2.11)	1.94 (1.48–2.38)	Z = 1.208	0.227
Platelet count (mmol/L)	233.00 (196.25–276.00)	233.00 (195.75–277.00)	236.00 (205.50–275.00)	Z = 0.591	0.554
NLR	2.33 (1.67–3.04)	2.38 (1.64–3.31)	2.27 (1.80–2.88)	Z = 0.772	0.440
PLR	128.11 (97.98–180.63)	129.51 (98.54–184.38)	124.41 (98.03–165.62)	Z = 0.368	0.713
PNR	56.77 (44.53–73.22)	55.05 (43.17–73.85)	59.02 (45.68–71.55)	Z = 0.866	0.386
Sex (%)				χ² = 2.353	0.125
Male	170 (82.52)	115 (79.86)	55 (88.71)		
Female	36 (17.48)	29 (20.14)	7 (11.29)		
Lymph node size (%)				χ² = 1.131	0.568
< 5mm	165 (80.1)	116 (80.56)	49 (79.03)		
5–10mm	27 (13.11)	17 (11.81)	10 (16.13)		
≥ 10mm	14 (6.8)	11 (7.64)	3 (4.84)		
TURBT grade (%)				χ² = 0.911	0.340
low grade	38 (18.45)	29 (20.14)	9 (14.52)		
high-grade	168 (81.55)	115 (79.86)	53 (85.48)		

BMI, body mass index; LNM, lymph node metastasis; NLR, neutrophil-to-lymphocyte ratio; PLR, platelet-to-lymphocyte ratio; PNR, platelet-to-neutrophil ratio; SD, standard deviation.

**Table 2:** Logistic regression analysis of the risk factors for lymph node metastasis.

Variables	Beta	SE	Z	OR (95% CI)	p	aBeta	aSE	aZ	aOR (95% CI)	aP
Age (years)	0.01	0.02	0.60	1.01 (0.97–1.06)	0.547					
Weight (kg)	-0.03	0.02	-1.21	0.97 (0.94–1.02)	0.228					
BMI (kg/m^2^)	0.03	0.02	1.14	1.03 (0.98–1.07)	0.255					
Tumor size (cm)	0.08	0.02	4.89	1.09 (1.05–1.12)	<.001	0.09	0.02	4.02	1.09 (1.05–1.14)	<.001
Serum calcium (mmol/L)	2.27	1.68	1.35	9.68 (0.36–259.76)	0.176					
Albumin (mmol/L)	-0.02	0.03	-0.82	0.98 (0.93–1.03)	0.415					
Neutrophil count (mmol/L)	0.02	0.07	0.30	1.02 (0.89–1.18)	0.761					
Lymphocyte count (mmol/L)	-0.60	0.35	-1.70	0.55 (0.27–1.10)	0.089					
Platelet count (mmol/L)	0.01	0.00	2.08	1.01 (1.01–1.01)	0.037	0.01	0.00	1.62	1.01 (1.00–1.02)	0.106
NLR	0.05	0.05	0.95	1.05 (0.95–1.17)	0.341					
PLR	0.01	0.00	2.42	1.01 (1.01–1.01)	0.015					
PNR	-0.00	0.01	-0.33	1.00 (0.98–1.01)	0.742					
Sex (%)										
male				1.00 (Reference)						
Female	-0.13	0.55	-0.23	0.88 (0.30–2.57)	0.816					
Lymph node size (%)										
< 5mm				1.00 (Reference)					1.00 (Reference)	
5–10mm	0.81	0.64	1.26	2.24 (0.64–7.84)	0.206	0.02	0.84	0.02	1.02 (0.20–5.29)	0.985
≥ 10mm	3.49	0.83	4.19	32.79 (6.42–167.47)	<.001	3.51	1.19	2.95	33.49 (3.24–346.49)	0.003
TURBT grade (%)										
low grade				1.00 (Reference)					1.00 (Reference)	
high-grade	2.10	1.04	2.02	8.18 (1.06–63.03)	0.044	2.32	1.10	2.10	10.15 (1.17–88.01)	0.035

CI, confidence interval; OR, odds ratio; SE, standard error.

**Figure 2:**
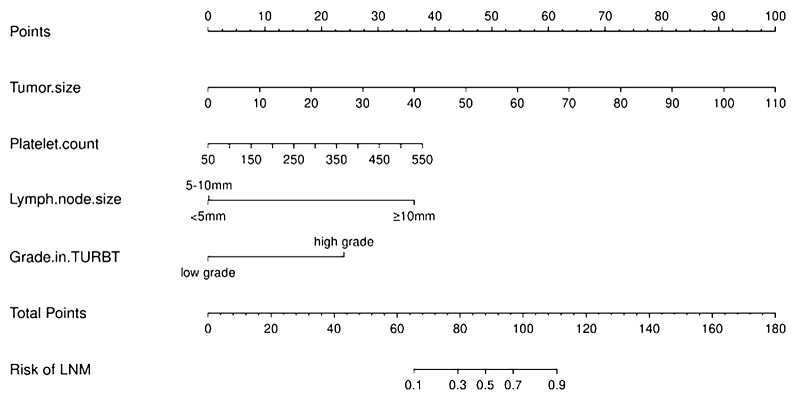
Nomogram for predicting lymph node metastasis in patients with progressing Muscle-invasive BC.

The risk of developing LNM increased with larger tumors, as indicated by preoperative imaging (odds ratio [OR] = 1.09, 95% confidence interval [CI]:1.05–1.14, p < 0.001). Tumor grading proved to be a significant independent predictor—LNM was more likely to occur in patients with higher-grade tumors compared with those with lower-grade ones (OR = 10.60, 95% CI:1.18–94.97, p < 0.05). Higher platelet counts were associated with an increased risk of LNM (OR = 1.01, 95% CI: (1.00–1.02), p = 0.106). Larger lymph nodes (as visualized on imaging) were more likely to be associated with LNM compared with smaller ones (≥ 10 mm vs. < 5 mm, OR = 35.82, 95% CI:3.37–308.53, p < 0.05; 5–10 mm vs. < 5 mm, OR = 1.11, 95% CI:0.19–6.55). We integrated these independent risk factors to establish a predictive model and established it as a nomogram (**Figure 2**).

### 3.2 Nomogram validation

**Figure 3:**
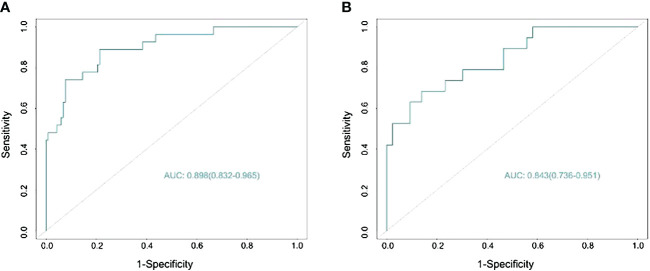
Predicting the area under the curve of lymph node metastasis (LNM) in patients with bladder cancer (BC) in the training **(A)** and validation **(B)** sets.

**Figure 4:**
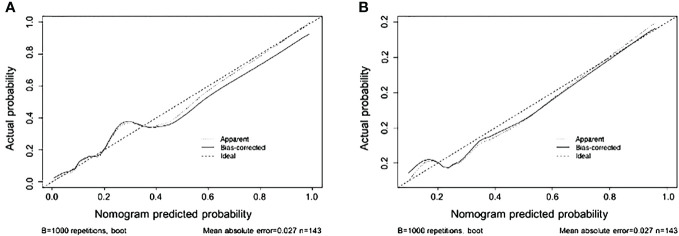
Nomogram calibration curves in the training **(A)** and validation **(B)** sets (bootstrap method using 1,000 repetitions).

The nomogram demonstrated an AUC of 0.898 (95% CI: 0.832–0.965) (**Figure 3A**) in the training cohort, which indicated that the nomogram effectively predicted the likelihood of LNM in patients with BC. The calibration curve (**Figure 4A**) exhibited a strong resemblance to the theoretical line, indicating that the nomogram had favorable characteristics of reproducibility and dependability. Upon utilization of the nomogram model generated internally, the validation cohort yielded an AUC of 0.843 (95% CI: 0.736–0.951) (**Figure 3B**), which indicated a substantial level of predictive accuracy in the external validation cohort. The calibration curve (**Figure 4B**) exhibited a strong resemblance to the ideal line, thereby confirming the satisfactory performance of the nomogram in the external validation cohort as well.

### 3.3 Clinical value of the nomogram

**Figure 5:**
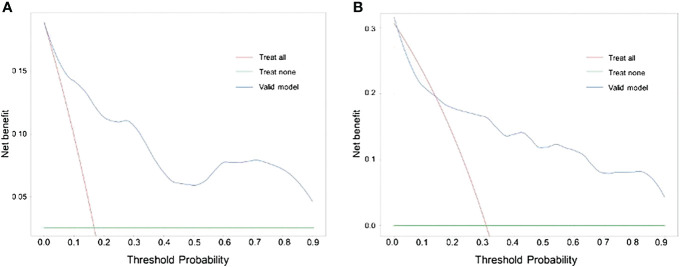
Nomogram decision curves in the training **(A)** and validation **(B)** set.

The findings of our DCA for predicting BC LNM indicated that the model had significant clinical advantages within the primary cohort throughout a threshold range of 0.05–0.90 (**Figure 5A**) and verified that the nomogram could have the potential to aid in the prediction of LNM in patients with BC within a threshold probability range of 0.15–0.90 (**Figure 5B**).

## 4 Discussion

Lymph nodes are the most common sites of metastasis in patients with BC (13). LNM has also been confirmed as an independent prognostic marker for BC survival. The ability to make preoperative predictions of LNM would facilitate better decisions on the choice between extended or standard LND in the context of bladder-preserving treatment or RC, depending on what is most beneficial in each patient with BC. Several studies have shown significant differences in disease-specific survival between patients with primary and those with progressive MIBC, with worse outcomes recorded in patients with MIBC who have prior histories of superficial BC (14). Therefore, there is significant clinical value in predicting LNM in patients with progressive MIBC.

RC with pelvic LND and urine diversion are the surgical interventions that are often performed in individuals diagnosed with MIBC, as well as in certain patients presenting with high-grade NMIBC (15). Patients with MIBC treated using RC alone, and those with BUC who have LNM, show a 5-year overall survival rate of only 19% (16). Appropriate treatment measures can prolong the survival rates of patients with BC and LNM (17). Previous studies have conducted comprehensive analyses on the impact of extended LND on the survival rates in patients diagnosed with BC and LNM (18). However, the complication rate of RC surgery is quite high, ranging between 62.4 and 85.7%, with 12.7–30% of patients experiencing severe complications (19). Certain patients with MIBC can also achieve quality-adjusted life years following bladder-preserving treatment (20). In some patients with high-grade NMIBC and a lower risk of progression and metastasis, RC may be considered an excessive treatment (21). Therefore, the preoperative prediction of LNM is crucial for optimal clinical decision-making.

Nomograms have the capability to give basic statistical analyses and visual representations of data, which can assist in clinical decision-making processes and foster the advancement of individualized medical interventions. The predictive model incorporated demographic patient variables, as previous research has indicated a negative correlation between age and the likelihood of LNM in patients with BC. For each 10-year increment in age, there was an approximate 20% drop in the probability of LNM (22). Obesity is another significant risk factor for adverse outcomes in patients with NMIBC (23). However, in our study, age and body mass index (BMI) were not independent risk factors for LNM, which may be linked to the different demographic characteristics of patients with progressive NMIBC and those with primary MIBC.

Traditional preoperative examinations often use CT and magnetic resonance imaging (MRI) to determine whether the abdominal and pelvic lymph nodes have been infiltrated by tumors (24). CT and MRI rely on morphological criteria such as lymph node size and shape to predict LNM (25). Conventionally, a short-axis lymph node diameter of 10 mm is the threshold for malignancy on CT and MRI (26). Consequently, This study constructed a precise nomogram for forecasting LNM in individuals diagnosed with BC. This nomogram was designed to integrate commonly used preoperative imaging markers with readily available clinical information. The results of our multifactorial logistic regression analysis indicate that lymph node and tumor sizes observed on imaging are independent risk factors for bladder urothelial carcinoma lymph node metastasis.

Patients with progressive MIBC often undergo TURBT before RC, and the proportion of patients with progressive MIBC who undergo secondary TURBT is higher than that of patients with general progressive MIBC (6). This helps improve the accuracy of histopathological diagnosis. Our univariate analysis showed that tumor grading was significantly higher in patients with BC and LNM compared with those without LNM. The multivariate logistic regression analysis conducted in our study revealed that tumor grade was a significant pathological risk factor for lymph node metastasis in BC. A prior investigation encompassing a cohort of 424 individuals diagnosed with breast cancer revealed a positive correlation between high-grade malignancies and the presence of LNM, consistent with our results (27).

Cantiello et al. confirmed that neutrophil-to-lymphocyte ratio (NLR) and platelet-to-lymphocyte ratio (PLR) are associated with the recurrence and progression of NMIBC, with worse oncological outcomes reported in patients with NMIBC with high NLRs and PLRs (28). Ziani et al. found that preoperative NLR values could predict the recurrence, progression, and failure of NMIBC treatment (29). Serum calcium level has also been confirmed to represent a risk factor for BC metastasis and lower pretreatment serum albumin levels have been associated with poorer outcomes following RC (30). In our study, easily measurable preoperative laboratory measurements such as serum calcium, albumin, neutrophil count, lymphocyte count, platelet count, NLR, PLR, and platelet-to-neutrophil ratio (PNR) were used as predictive indicators of LNM in cases of progressive MIBC, with platelet count ultimately being determined to be an independent risk factor. Platelets are critical regulators of lymphatic angiogenesis, aiding both physiological and pathological lymphatic vessel growth by releasing angiogenic factors from specialized alpha granules (31) and are involved in tumor angiogenesis, supporting tumor growth by promoting vascular stability and angiogenesis (32). This may explain why platelet count is an independent risk factor for LNM in cases of progressive MIBC. These effects occur despite our univariate analysis showing significant differences between progressive MIBC LNM and non-LNM MIBC—whereas our multivariate logistic regression analysis found no such differences between the two. These contradictory findings suggest that these biomarkers are controversial and require further analysis in studies with larger sample sizes.

Consequently, the nomogram developed in this study could be a comprehensive tool for preoperatively predicting the likelihood of lymph node metastasis in patients diagnosed with BC. This nomogram incorporates variables such as platelet count, and tumor size as determined by imaging, lymph node size, and tumor pathology grading. To ascertain the clinical significance of the nomogram, we incorporated a comprehensive set of pathological, laboratory, and imaging data from TURBT specimens that are representative and readily attainable. The nomogram showed an AUC of 0.898 (95% CI: 0.832–0.965) in the training cohort, and 0.843 (95% CI: 0.736–0.951) in the external validation cohort, suggesting a favorable level of predictive accuracy. The calibration curves for both the main and validation cohorts demonstrated strong performance throughout the process of internal and external validations. Furthermore, the results of the DCA analysis for both cohorts exhibited a significant degree of clinical applicability.

This study has some limitations. The sample size was limited, which may have affected the generalizability of the findings. Additionally, our internal and external cohorts were derived from the same center. Therefore, additional multi-center investigations remain warranted. Furthermore, among the patients screened based on the inclusion and exclusion criteria, only six were pathologically diagnosed with carcinoma *in situ* (CIS). Including CIS in the study would introduce selection bias due to our insufficient data, thus CIS was not considered as a covariate. To address the above-mentioned issues, we will conduct multicenter studies with large sample sizes in the future to refine the predictive models based on this study. Furthermore, we will validate the clinical efficacy of the predictive models through prospective research.

## 5 Conclusion

We developed a nomogram using pathological features from TURBT specimens, imaging data, and laboratory test indicators to predict LNM in patients with progressive MIBC. Following internal and external validation, this nomogram showed high accuracy, reliability, and clinical applicability. Our results have potentially positive implications in the clinical treatment of this vulnerable patient group.

## Data availability statement

The raw data supporting the conclusions of this article will be made available by the authors, without undue reservation.

## Ethics statement

The studies involving humans were approved by Ethics Committee, The Affiliated Hospital of Qingdao University. The studies were conducted in accordance with the local legislation and institutional requirements. Written informed consent for participation was not required from the participants or the participants’ legal guardians/next of kin in accordance with the national legislation and institutional requirements.

## Author contributions

YQ: Investigation, Software, Writing – original draft. YJ: Data curation, Writing – original draft. LL: Methodology, Writing – original draft. BL: Resources, Writing – original draft. FX: Validation, Writing – review & editing. HW: Formal analysis, Methodology, Writing – review & editing. SL: Supervision, Writing – review & editing.
